# Author Correction: Caveolae-mediated Tie2 signaling contributes to CCM pathogenesis in a brain endothelial cell-specific *Pdcd10*-deficient mouse model

**DOI:** 10.1038/s41467-025-61617-0

**Published:** 2025-07-10

**Authors:** Huanjiao Jenny Zhou, Lingfeng Qin, Quan Jiang, Katie N. Murray, Haifeng Zhang, Busu Li, Qun Lin, Morven Graham, Xinran Liu, Jaime Grutzendler, Wang Min

**Affiliations:** 1 Interdepartmental Program in Vascular Biology and Therapeutics, Department of Pathology, Yale University School of Medicine, New Haven, CT USA; 2 Department of Neurobiology, Yale University School of Medicine, New Haven, CT USA; 3 Department of Cell Biology, Yale University School of Medicine, New Haven, CT USA

**Keywords:** Cell signalling, Mechanisms of disease, Vascular diseases

Correction to: *Nature Communications* 10.1038/s41467-020-20774-0, published online 25 January 2021

In the version of the article initially published, Fig. 2b contained the wrong image for “P21 deletion (no lesion)” and has been corrected. In the top row of Fig. 5l, the “siCT” image was inadvertently a duplicate of the “siCav1” image and has been corrected. In the top row of Fig. 6b, the “WT” image was a “Cav1KO, HE” image and has been corrected. In Supplementary Fig. 1f, a “Pdcd10becKO” spleen image was mistakenly used in place of the “WT” image which has now been corrected. In the “Cerebellum” row of Supplementary Fig. 9i, the “WT + IgG” image was a “WT + Angpt2” image and has been corrected. In Supplementary Fig. 10b, the “SMA”, “WT + Rebastinib” image was incorrect and has been amended. These corrections have been made to the HTML and PDF versions of the article. The original figures are available for comparison in the Supplementary information accompanying this amendment.

## Supplementary information

Original Figs. 2, 5 and 6 and Supplementary Figs. 1, 9 and 10

