# Supplementary material for: Caveolae-mediated Tie2 signaling contributes to CCM pathogenesis in a brain endothelial cell-specific Pdcd10-deficient mouse model
Source: Nat Commun. 2021 Jan 25;12:504. doi: 10.1038/s41467-020-20774-0 (PMC7835246; doi:10.1038/s41467-020-20774-0)
Supplement: Supplementary file 1 — Supplementary Information [file 41467_2020_20774_MOESM1_ESM.pdf]

SUPPLEMENTARY FIGURE AND LEGENDS

Supplementary Figure 1 *Pdcd10*^{ECKO} mice exhibit severe splenic vascular defects.

Supplementary Figure 2 Mfsd2a-CreER^{T2} drives a brain and retinal EC-specific gene deletion.

Supplementary Figure 3 Venous malformations at the periphery of the retinal vascular plexus.

Supplementary Figure 4 *Pdcd10*^{BECKO} mice exhibit no lesion in other tissues outside brain and retina.

Supplementary Figure 5 EM and tomography for increased caveolae in *Pdcd10*^{BECKO} brain ECs.

Supplementary Figure 6 Protein expression in mouse cerebrum and cerebellum.

Supplementary Figure 7 Supplementary data for Fig.5 (CCM3 loss augments Cav1-Tie2 signaling).

Supplementary Figure 8 Cav1-deletion rescue CCM lesions in retina.

Supplementary Figure 9 Tie1 correlates with Angpt2-Tie2 activation in *Pdcd10*^{BECKO} cerebrum tissues and Angpt2-neutralizing antibody therapeutics in *Pdcd10*^{BECKO} mice.

Supplementary Figure 10 Tie2 inhibitor inhibits Tie2 activation and reduces α-SMA expression.

Supplementary Figure 11 Specific effects of Rebastinib on Tie2.

Supplementary Table 1: Antibodies for immunostaining.

Supplementary Table 2: Primers for qRT-PCR.

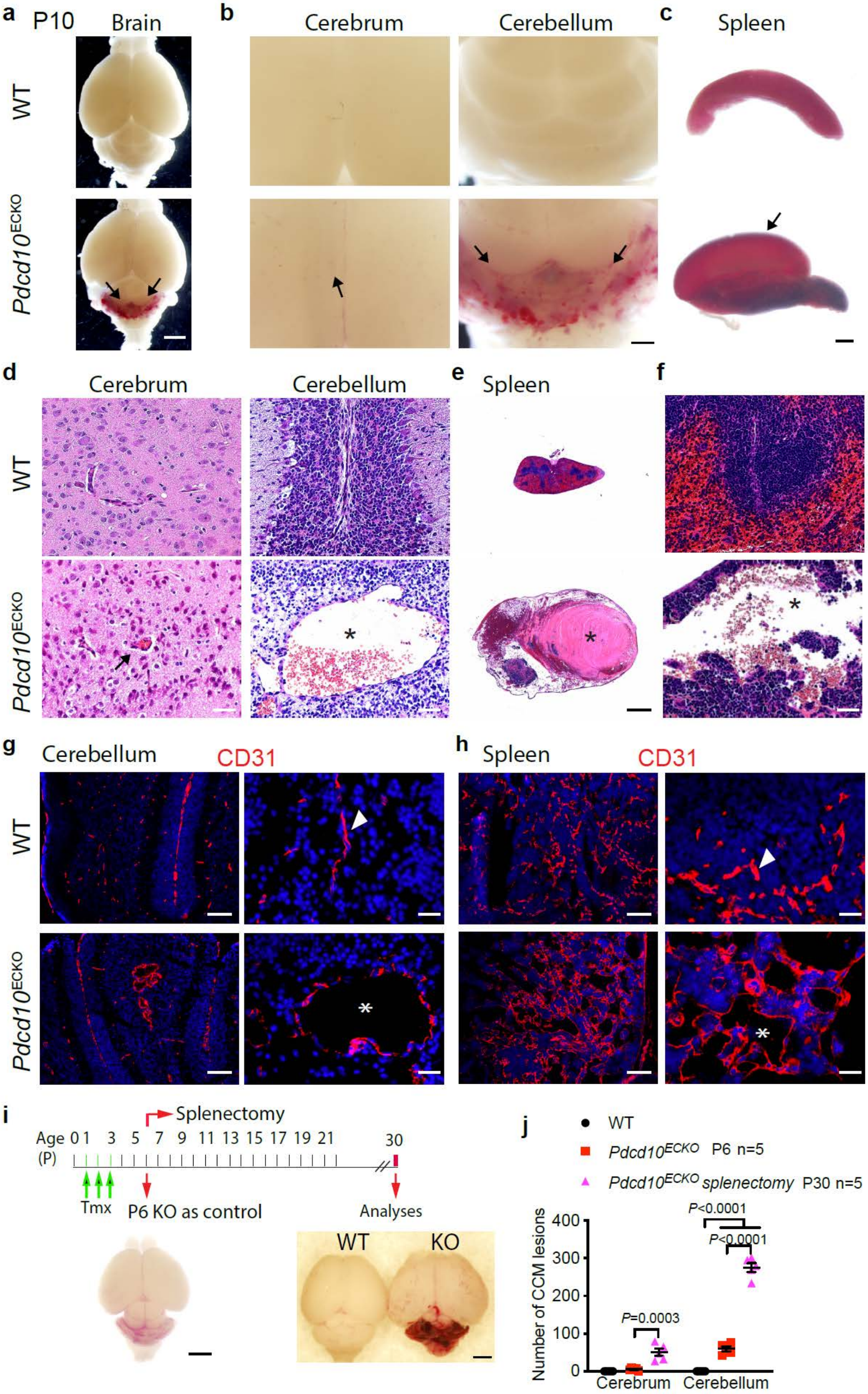
a
P10
Brain
WT
Pdcd10^ECKO
b
Cerebrum
Cerebellum
c
Spleen
d
Cerebrum
Cerebellum
e
Spleen
f
g
Cerebellum
CD31
h
Spleen
CD31
i
Splenectomy
Age 0 1 3 5 7 9 11 13 15 17 19 21 30
(P)
Tmx
P6 KO as control
Analyses
WT
KO
j
WT
Pdcd10^ECKO P6 n=5
Pdcd10^ECKO splenectomy P30 n=5
Number of CCM lesions
P<0.0001
P<0.0001
P=0.0003
Cerebrum
Cerebellum

Supplementary Fig.1. *Pdcd10*^{ECKO} mice exhibit severe splenic vascular defects. Tissues from WT and *Pdcd10*^{ECKO} pups were harvested at P10 after P1 deletion by tamoxifen injection. Fresh tissues (**a-c**), sections stained with H&E (**d-f**), and immunostaining of the vasculature with CD31 (**g-h** with high magnification images on the right) are shown. Lesions are indicated by arrows (fresh tissue) and asterisks (H&E and immunostaining), whereas normal WT vessels are indicated by arrowheads. **i-j**. *Pdcd10*^{ECKO} pups were fed with tamoxifen at P1 to P3; at P6 one group of mice were subjected to splenectomy and harvested at P30. Representative brain images for P6 and P30 mice are shown (i). Number of CCM lesions were quantified as # of lesions per 10 coronal sections, which were 200 µm apart. n = 5 mice per group. *P* values are indicated, one-way ANOVA followed by Sidak's multiple comparisons test. Data are means ± SEM. Scale bars: 2 mm (a,i); 500 µm (b, c); 400 µm (e); 100 µm (left panel in g and h); 25 µm (d, f and right panel in g and h). Source data are provided as a Source Data file.

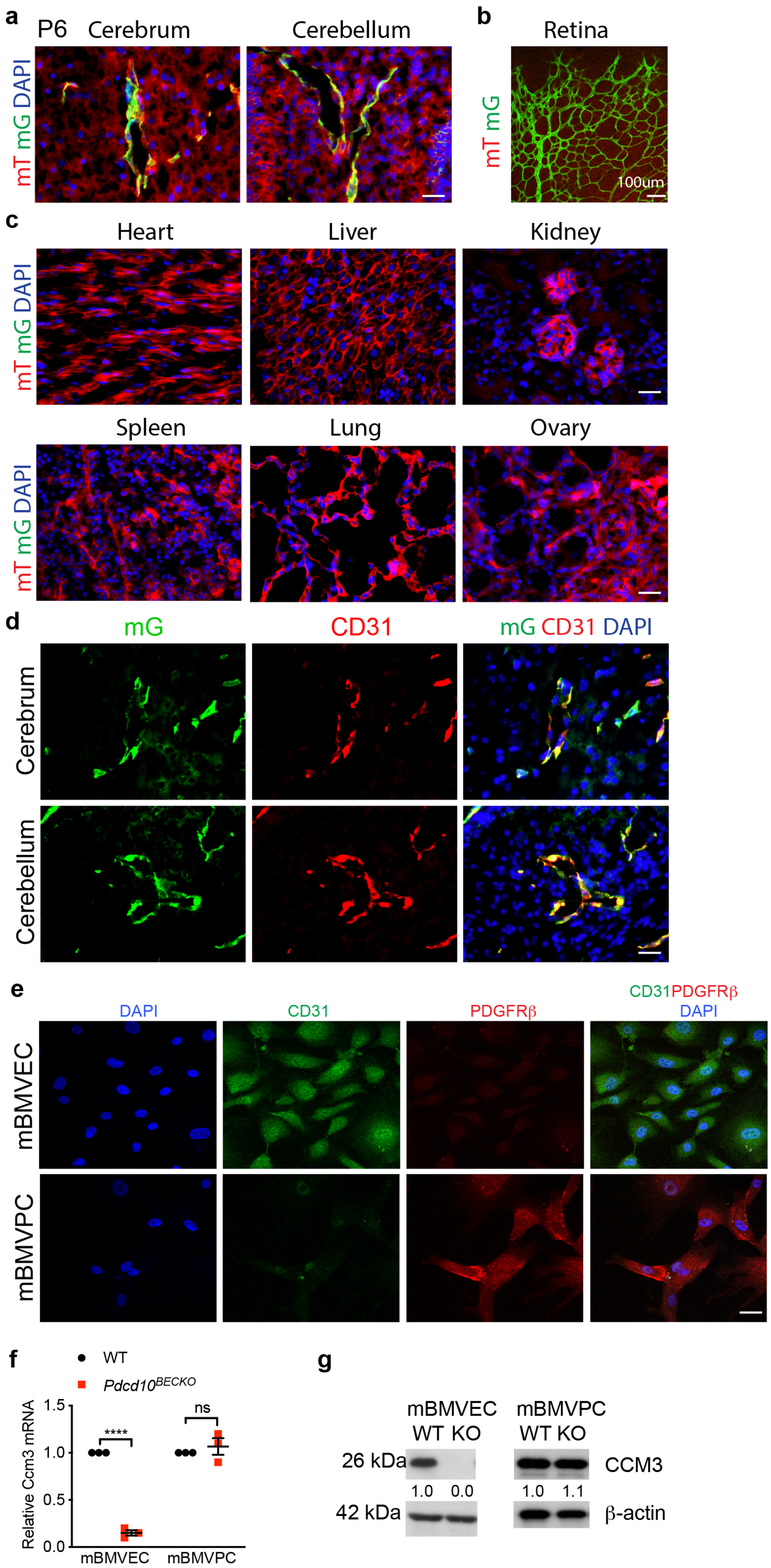
a
P6 Cerebrum
Cerebellum
mT mG DAPI
b
Retina
mT mG
100um
c
Heart
Liver
Kidney
Spleen
Lung
Ovary
d
mG
CD31
mG CD31 DAPI
Cerebrum
Cerebellum
e
DAPI
CD31
PDGFRβ
CD31PDGFRβ DAPI
mBMVEC
mBMVPC
f
WT
Pdcd10BECKO
Relative Ccm3 mRNA
1.5
1.0
0.5
0.0
****
ns
mBMVEC
mBMVPC
g
mBMVEC
WT KO
mBMVPC
WT KO
26 kDa
CCM3
1.0 0.0
1.0 1.1
42 kDa
β-actin

Supplementary Fig.2. Mfsd2a-CreERT2 drives a brain and retinal EC-specific gene deletion.

a-c. mT/mG reporter mice were bred with Mfsd2a-CreERT2;*Pdcd10*$^{fl/fl}$ deleter mice followed by tamoxifen feeding from P1 to P3. Tissues were harvested at P6 and mT/mG were examined. mG expression was specifically detected in the vasculature of brain sections and retina (whole-mount), but not in other tissues. *n*=3 mice per group. **d**. The brain sections were further immunostained with endothelial cell marker CD31. mG expression was completely co-localized with CD31 staining in both the cerebrum and cerebellum. *n*=3 mice per group. **e.** Mouse brain microvessel ECs (mBMVECs) and mouse brain microvessel pericytes (mBMVPCs) were isolated from WT mice at P6, and immunostained with EC marker CD31 and pericyte marker PDGFRb. Representative immunofluorescence images are presented. **f-g.** *Ccm3* deletion was specifically in mouse brain ECs but not in mouse brain pericytes. mBMVPCs and mBMVECs were isolated from P6 WT and *Pdcd10*^{BECKO} pups. *Ccm3* gene expression was determined by qRT-PCR. n = 3; *P* values are indicated, Unpaired two-tailed Student's *t*-test. Data are means ± SEM. unpaired two-tailed Student's *t*-test. (g) CCM3 protein was determined by western blotting. Representative blot from three experiments. Scale bars: 25 μm (a, c, d, e); 100 μm (b). Source data are provided as a Source Data file.

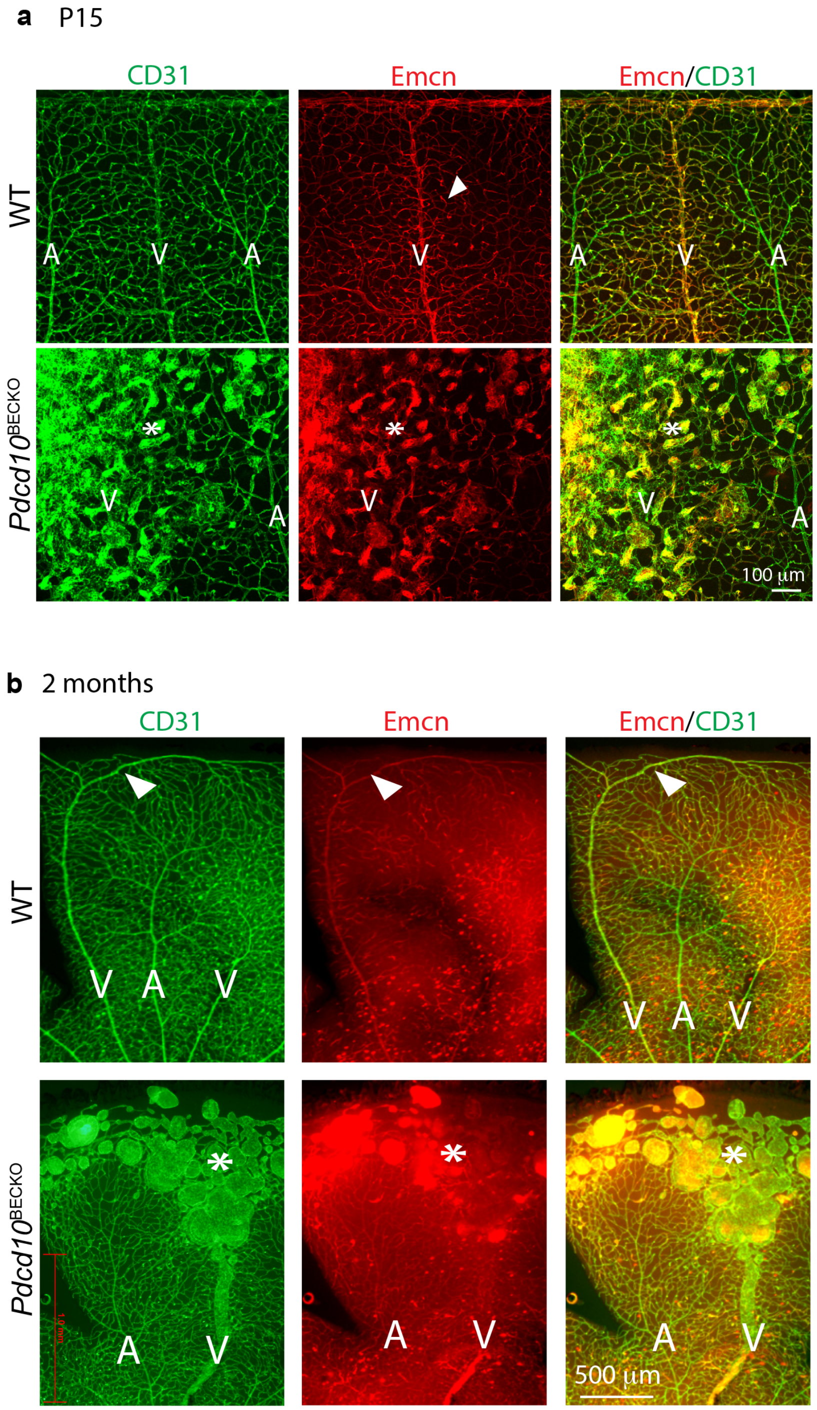

Supplementary Fig.3. ***Pdcd10*$^{\mathbf{BECKO}}$ mice develop venous malformations at the periphery of the retinal vascular plexus**. (**a**) Retinas from WT and *Pdcd10*^{BECKO} pups at P15 were stained with the pan-endothelial cell marker CD31 (green) and venous EC marker endomucin (Emcn). (**b**) Retinas from WT and *Pdcd10*^{BECKO} pups at P60 were stained with endothelial cell marker CD31 (green) and venous EC marker endomucin (Emcn). Artery (A) and vein (V) are marked. Arrowheads indicate normal vessels and asterisks indicate CCM lesions with a disorganized vasculature. *n*=6. Scale bar: 100 µm (a), 500 µm (b). Source data are provided as a Source Data file.

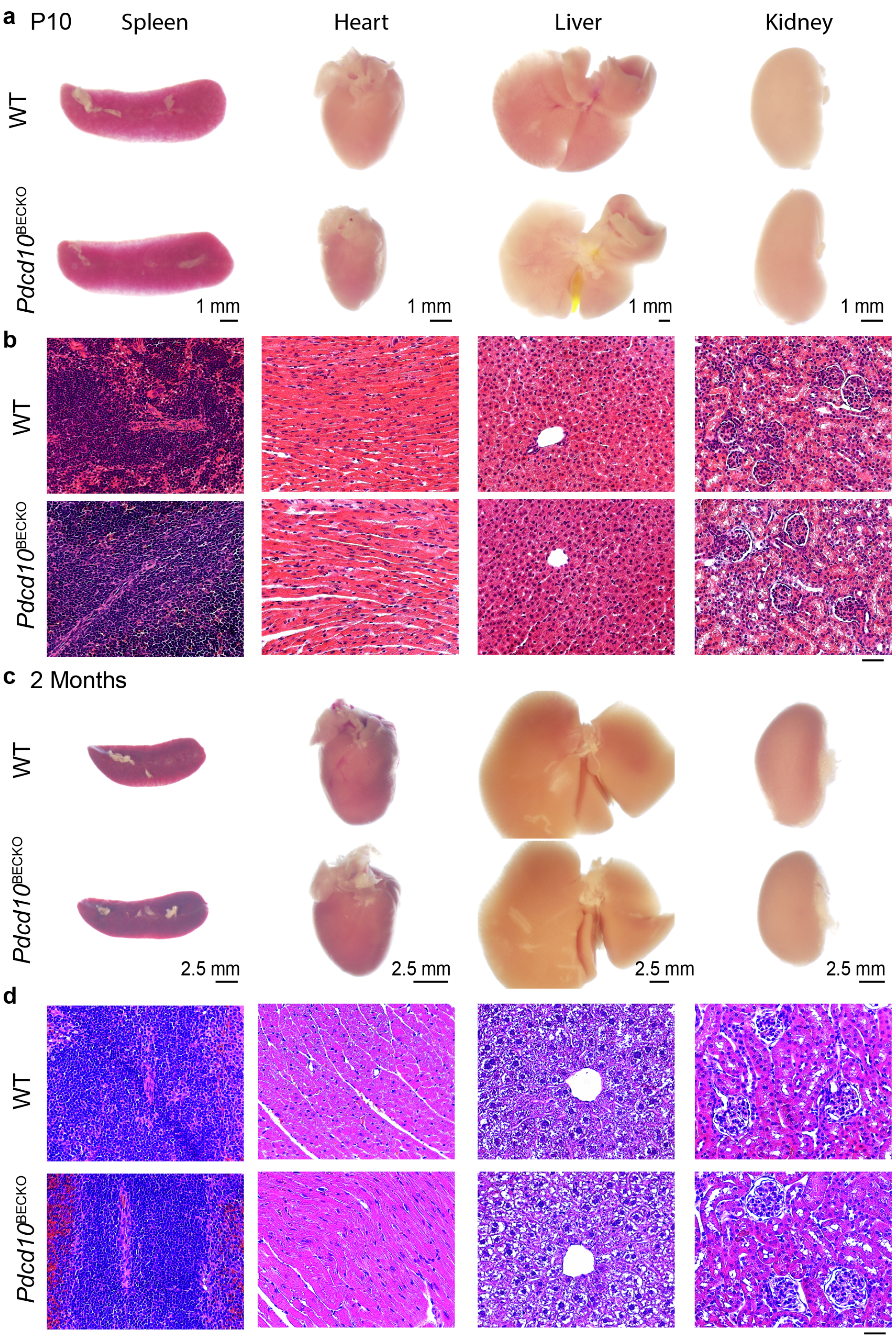

Supplementary Fig.4. *Pdcd10*^{BECKO} mice exhibit no lesion in other tissues outside brain and retina.
WT and *Pdcd10*^{BECKO} pups were fed with tamoxifen from P1 to P3, and mouse tissues were harvested at P10 and 2 months. Representative images of fresh tissues (**a, c**) and H&E staining (**b, d**) are shown. *n*=6. Scale bars: 1 mm (a, fresh tissues); 2.5 mm (c, fresh tissues); 25 µm (b,d; H&E). Source data are provided as a Source Data file.

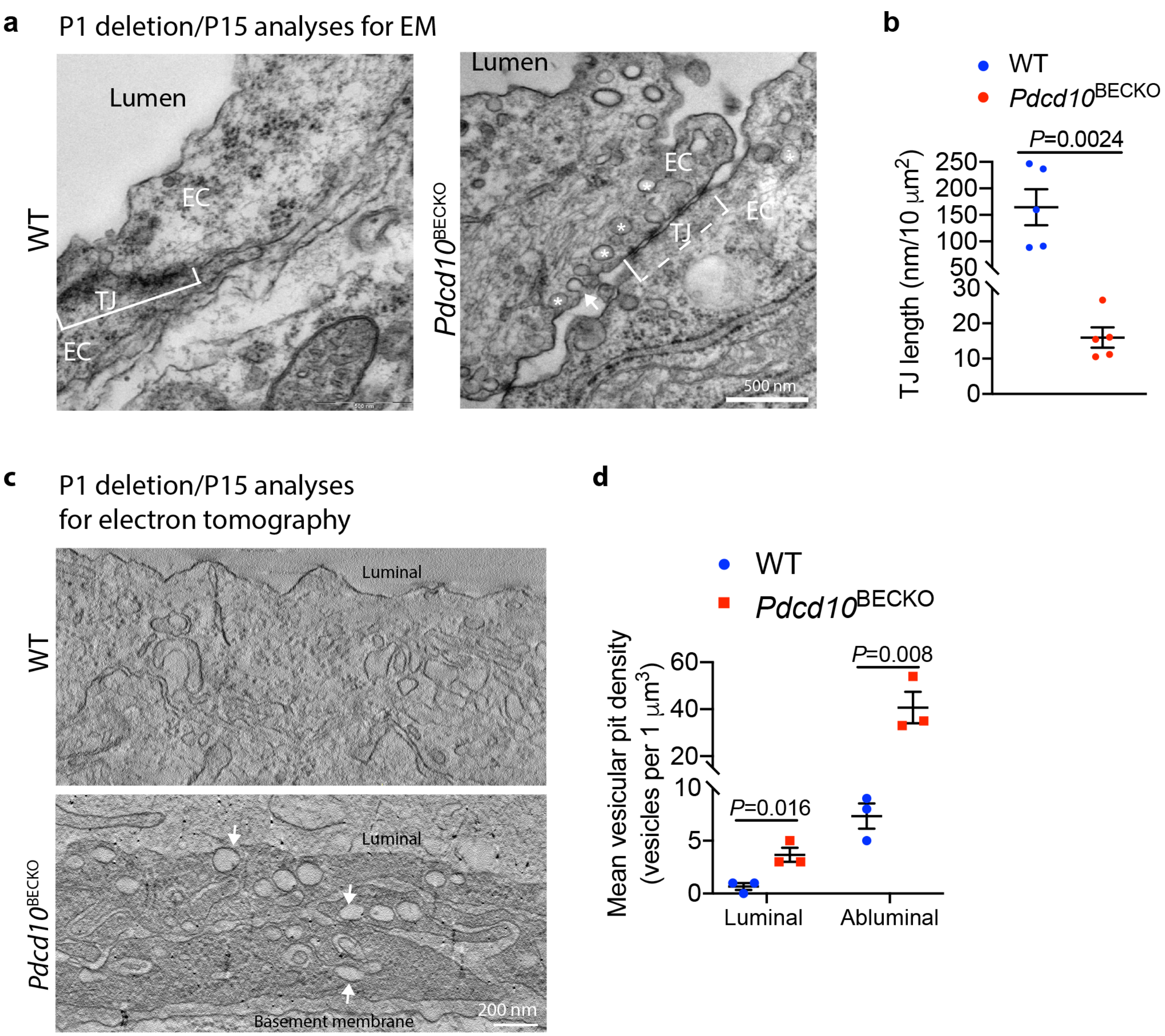

Supplementary Fig.5. Increased caveolae in *Pdcd10*^{BECKO} brain ECs determined by EM and electron tomography. Cerebral sections from WT and *Pdcd10*^{BECKO} mice (P1 deletion/P15 harvest) were subjected to EM (**a-b**) and electron tomography (**c-d**). (a) Representative EM images from 5 mice are shown. (a) Tight junction (TJ) was indicated by bracket in WT and dashed bracket in *Pdcd10*^{BECKO}. Caveolae was indicated by asterisks. (b) TJ length was quantified per 10 mm^2. 20 EM section per mouse and n=5. (c) Representative electron tomography images from 3 mice are shown. 120 layers with 2 nm per layer were scanned. Luminal and basement membrane are indicated. Representative luminal, intracellular and abluminal caveolae are labeled by arrows. (d) Mean vesicular pit density at luminal and abluminal sides are quantified per 1 mm^3. n=3. Unpaired two-tailed Student's *t*-test. Data are means ± SEM. Scale bars: 500 nm (a), 200 nm (c). Source data are provided as a Source Data file.

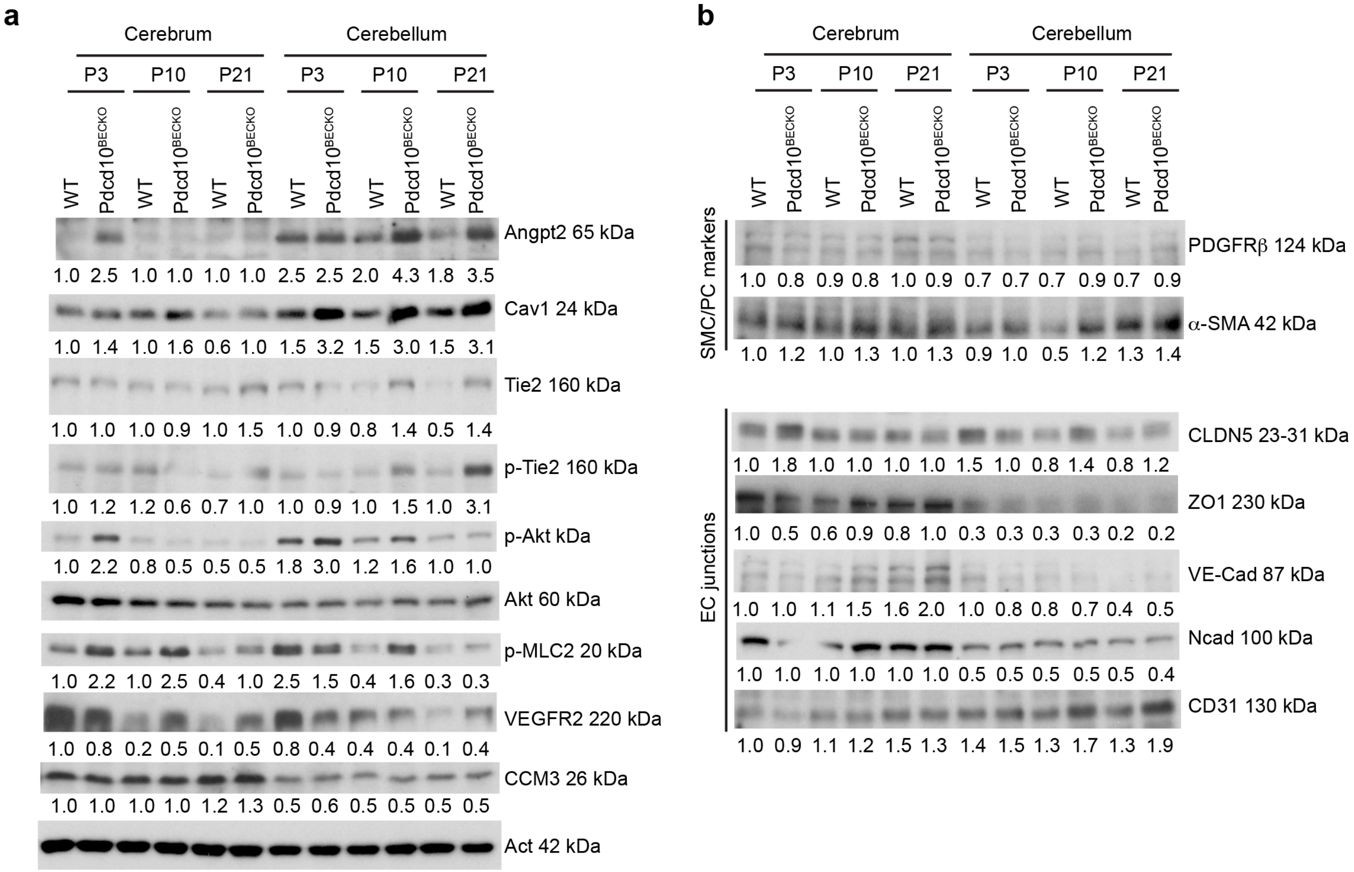

Supplementary Fig.6. Protein expression in mouse cerebrum and cerebellum. WT and P1 deletion *Pdcd10*BECKO brain tissues were harvested at various ages (P3, P10 and P21). **a**. Brain lysates were subjected to Western blotting with respective antibodies involved in Cav1-Tie2 and VEGFRs signaling. **b**. Brain lysates were subjected to Western blotting with respective antibodies involved in pericyte markers and EC junctions. The samples derived from the same experiment and all gels/blots were processed in parallel. Protein fold changes in both a and b are indicated by taking P3 WT cerebrum as 1.0. n=2. Act: β-actin; Cldn5: claudin-5; MLC2: myosin light chain-2; Ncad: N-cadherin; α-SMA: α -smooth muscle actin; VE-cad: VE-cadherin; ZO-1: zona occludens protein-1. Source data are provided as a Source Data file.

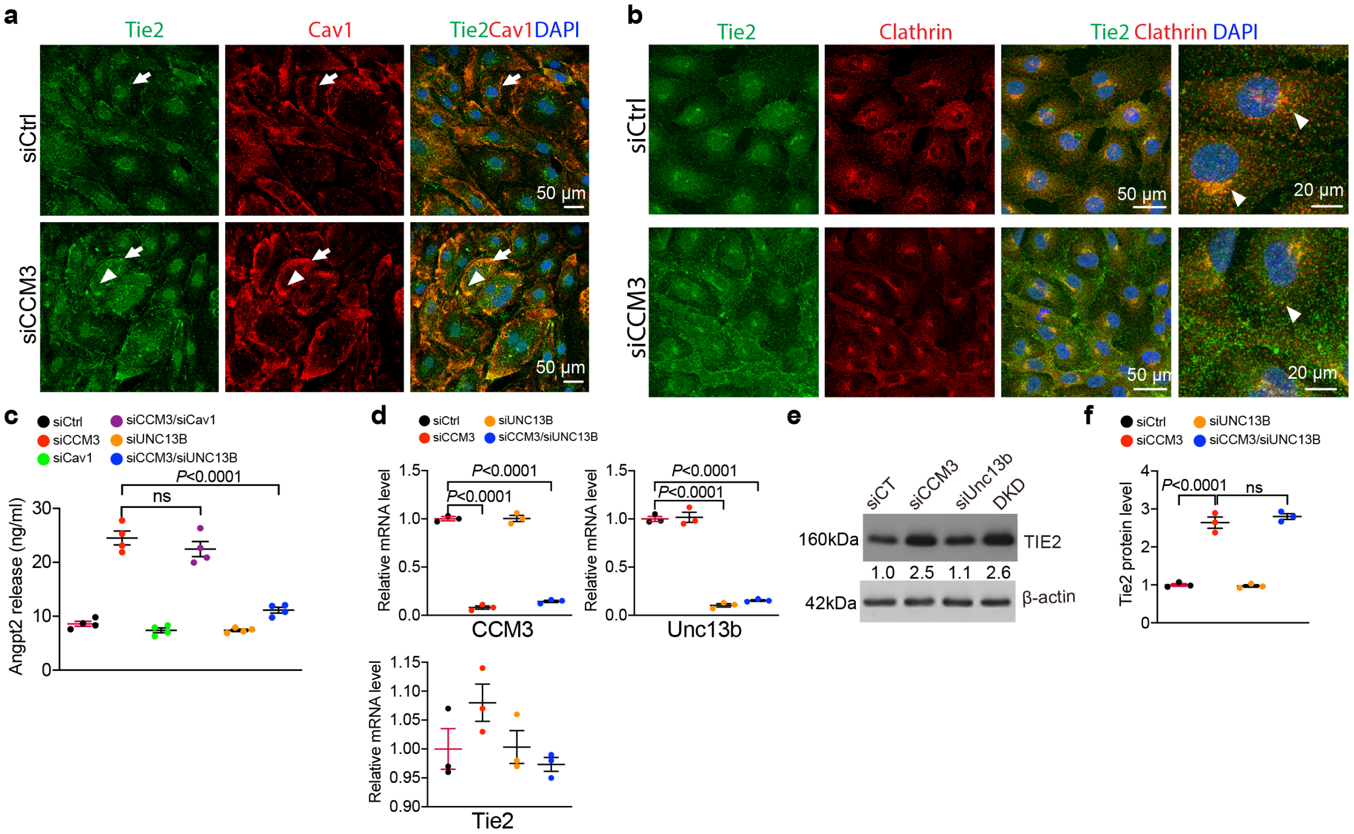

Supplementary Fig.7. Supplementary data for Fig.5 in vitro studies.

a. Co-localization of Tie2 and Cav1. HBMVECs were transfected with Ctrl siRNAs or CCM3 siRNAs for 72 h. Ctrl siRNA and CCM3 siRNA-transfected cells were subjected to immunofluorescence staining by co-staining with Tie2 (green) and Cav1 (red) with DAPI counterstaining (blue). Arrows indicate Tie2 membrane localization while arrowhead for intracellular Tie2. **b.** Colocalization of Tie2 and clathrin was not increased in CCM3-deficient cells. siRNA-transfected ECs were subjected to immunofluorescence staining by co-staining with Tie2 (green) and clathrin (red) with DAPI counterstaining (blue). **c.** Cav1 is not involved in Angpt2 secretion. The cells were transfected with indicated siRNAs for 72 h. The transfected cells were replenished with fresh media and Angpt2 accumulated in media between 0 and 8 h were determined by ELISA. *n*=4. **d-f.** Co-silencing of Unc13B did not attenuate Tie2 expression in CCM3-deficient ECs. HBMVECs were transfected with Ctrl siRNAs, CCM3 siRNAs, Unc13b siRNA or co-silencing of CCM3 and Unc13b for 72 h. **d**. Tie2 mRNA was not altered by CCM3 siRNA or Unc13b siRNA. mRNAs were measured by qRT-PCR with specific primers with 18S rRNA as an internal control. Relative mRNA levels were quantified and fold changes are presented by taking siCtrl as 1.0. n=3. **e-f.** Tie2 protein was not attenuated by co-silencing of Unc13b. Representative gel is shown in e. (f) Relative Tie2 protein levels were quantified and fold changes are presented by taking siCtrl as 1.0. n=3. Data are means ± SEM. *P* values are indicated, one-way ANOVA followed by Tukey's multiple comparisons test (c, d). Scale bars: 50 µm (a; b left three panels); 20 µm (b right panel). Source data are provided as a Source Data file.

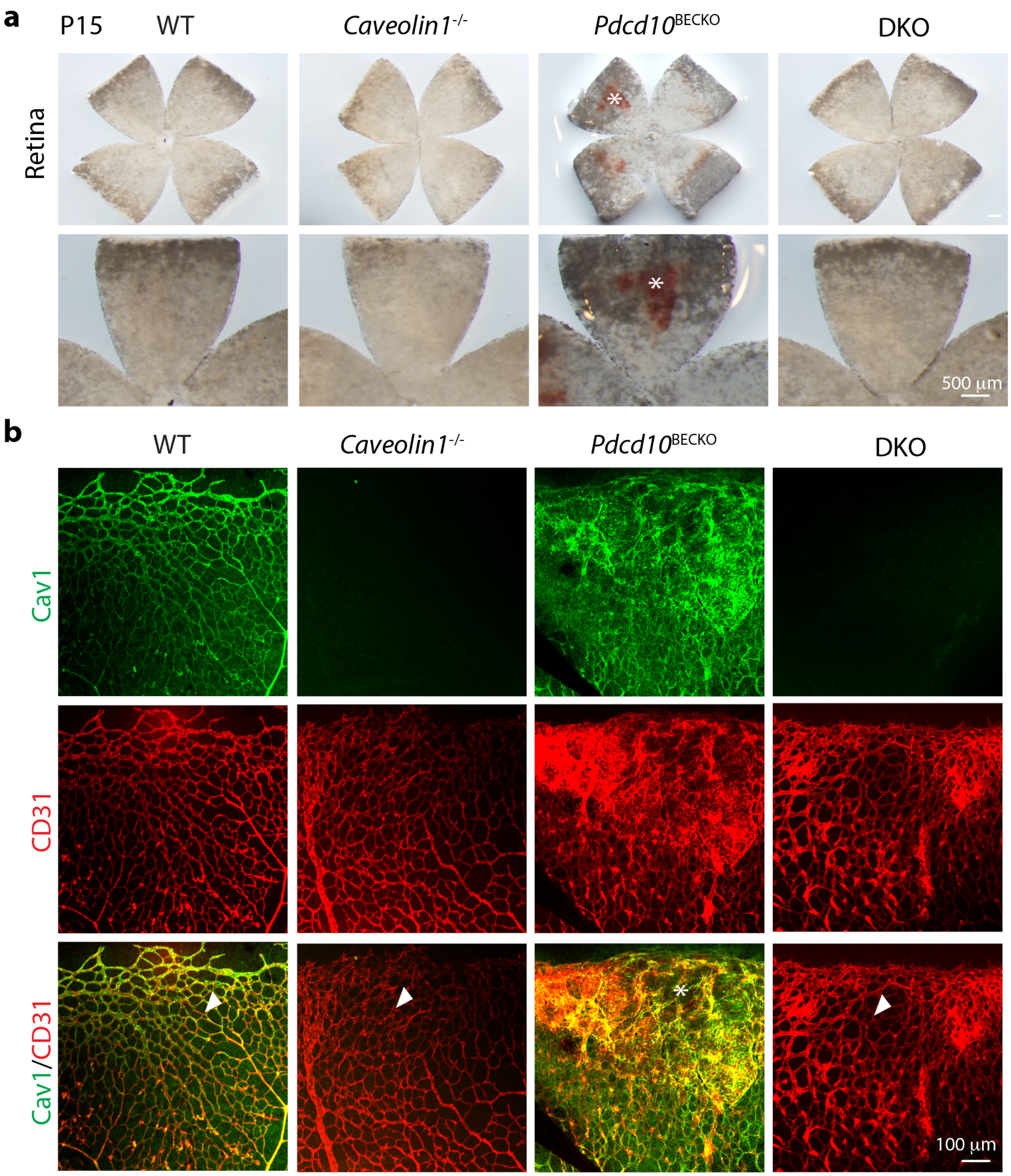

Supplementary Fig.8. Cav1-deletion rescues retinal CCM lesions in *Pdcd10*^{BECKO} mice. Ccm3$^{lox/lox}$ (WT), *Cav*$^{-/-}$ (*Cav1*-KO), *Pdcd10*^{BECKO} *and Pdcd10*^{BECKO}:*Cav*$^{-/-}$ (DKO) pups were fed with tamoxifen from P1 to induce deletion of *Ccm3*. Retinas were harvested at P15, and CCM lesions were visualized by fresh tissue imaging (a) and CD31 immunostaining (b). **a**. Images for fresh retinal tissues. Representative images are shown where lesions are indicated asterisks. **b**. Co-staining for CD31/Cav1. Representative images of normal vessels (arrowheads) and CCM lesions (asterisks) are shown. n=5, scale bars: 500 µm (a); 100 µm (b). Source data are provided as a Source Data file.

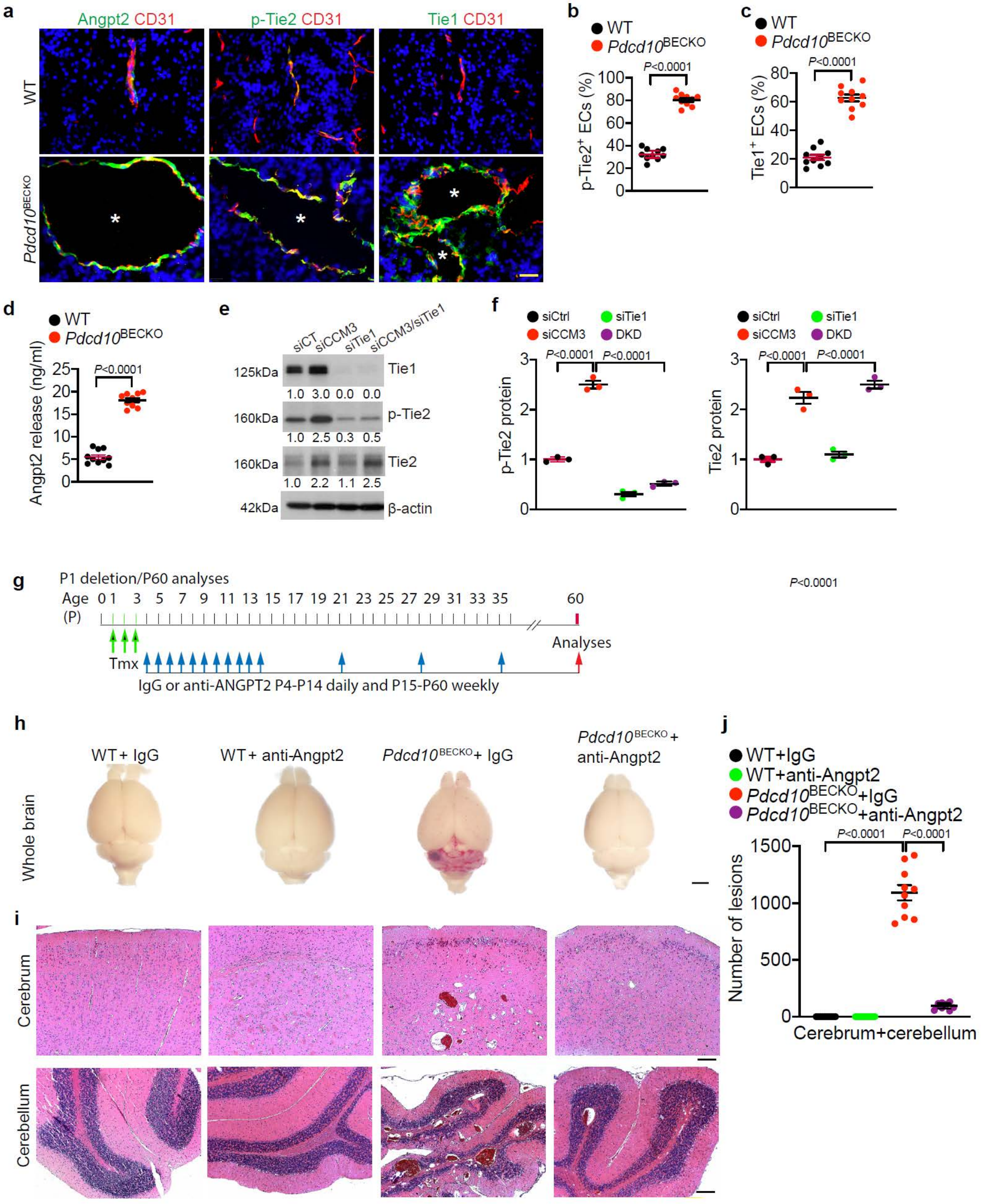
a
Angpt2 CD31
p-Tie2 CD31
Tie1 CD31
WT
Pdcd10BECKO
b
WT
Pdcd10BECKO
P<0.0001
p-Tie2+ ECs (%)
c
WT
Pdcd10BECKO
P<0.0001
Tie1+ ECs (%)
d
WT
Pdcd10BECKO
P<0.0001
Angpt2 release (ng/ml)
e
siCT siCCM3 siTie1 siCCM3/siTie1
125kDa Tie1
1.0 3.0 0.0 0.0
160kDa p-Tie2
1.0 2.5 0.3 0.5
160kDa Tie2
1.0 2.2 1.1 2.5
42kDa β-actin
f
siCtrl siTie1
siCCM3 DKD
P<0.0001 P<0.0001
p-Tie2 protein
Tie2 protein
g
P1 deletion/P60 analyses
Age (P)
Tmx
Analyses
IgG or anti-ANGPT2 P4-P14 daily and P15-P60 weekly
P<0.0001
h
WT+ IgG
WT+ anti-Angpt2
Pdcd10BECKO+ IgG
Pdcd10BECKO + anti-Angpt2
Whole brain
i
Cerebrum
Cerebellum
j
WT+IgG
WT+anti-Angpt2
Pdcd10BECKO+IgG
Pdcd10BECKO+anti-Angpt2
P<0.0001 P<0.0001
Number of lesions
Cerebrum+cerebellum

Supplementary Fig.9. Tie1 correlates with Angpt2-Tie2 activation in *Pdcd10*$^{\mathbf{BECKO}}$ cerebrum tissues and Angpt2-neutralizing antibody therapeutics in *Pdcd10*$^{\mathbf{BECKO}}$ mice.

(**a-c**) Tamoxifen injection (P1-P3) and mice were sacrificed and analyzed at age of P15. Co-staining for Angpt2, p-Tie2 or Tie1 with CD31. (**a**) Representative merged images of normal vessels and CCM lesions (asterisks) are shown. (**b-c**) Quantification of p-Tie2- and Tie1-positive ECs, i.e., % green (p-Tie2 or Tie1)-conjugated area/total red (CD31) cells by Image J. Arrowheads indicate normal vessels and asterisks indicate lesions. (**d**) Tamoxifen injection (P1-P3) and mice were sacrificed and analyzed at age of P15. Brain tissues were collected and Angpt2 levels were determined by ELISA. All Data are means ± SEM, n=10, P values are indicated (P <0.0001, Unpaired two-tailed Student's *t*-test). **e-f.** Tie1 was increased in CCM3-KO ECs. (e) Various siRNA-transfected hBMVECs cells were subjected to Western blotting for Tie1 and p-Tie2. (f) Relative total Tie2 and p-Tie2 protein levels were quantified and fold changes are presented by taking Ctrl siRNA as 1.0. n=3. P values are indicated, one-way ANOVA followed by Tukey's multiple comparisons test. **g.** Timeline of tamoxifen injection and anti-Angpt2 administration. Tamoxifen injection (P1-P3) followed by administration of an Angpt2-neutralizing antibody or IgG control (P4-P14 daily and P15-P60 weekly). Mice were sacrificed and analyzed at P60 (2 months). **h.** Images of fresh brain tissue from WT and *Pdcd10*^{BECKO} mice. **i-j**. H&E staining (i) and lesion quantifications in cerebrum and cerebellum (j). n=10, P values are indicated, one-way ANOVA followed by Tukey's multiple comparisons test. All Data are means ± SEM. Scale bars: 25 μm (a); 2 mm (h); 100 μm (i). Source data are provided as a Source Data file.

a P1 deletion and analyses on P15

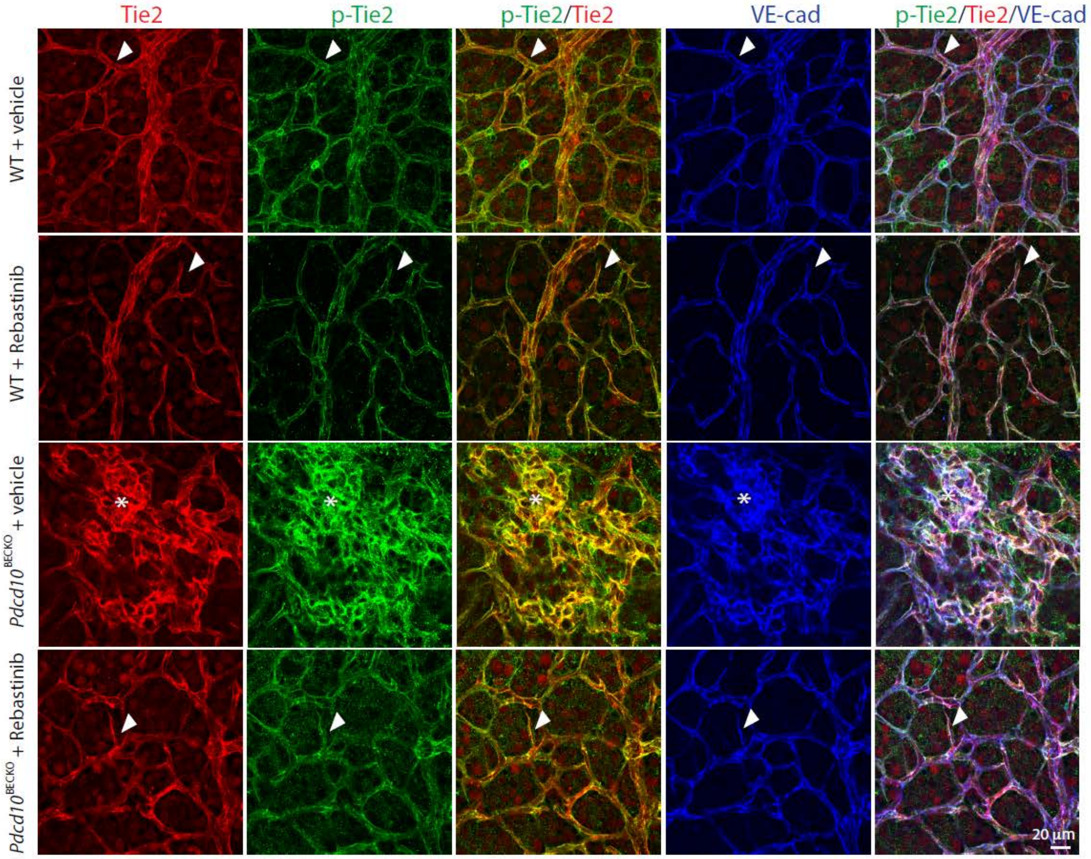

b P1 deletion and analyses on P15

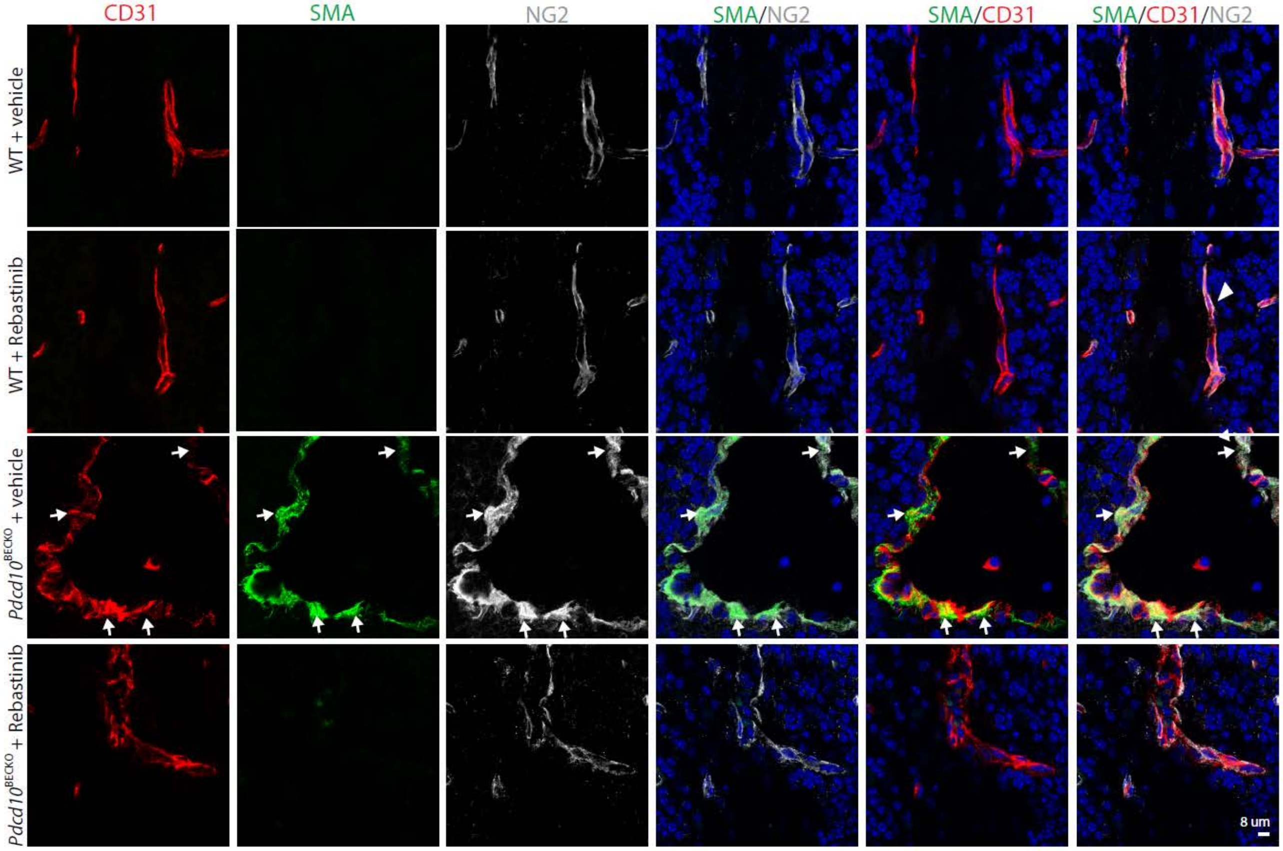

Supplementary Fig.10.

a. Tie2 inhibitor Rebastinib attenuates Tie2 signaling and CCM lesions in retina. Vehicle or Rebastinib was subcutaneously injected into Ctrl and *Pdcd10*^{ECKO} mice from P4 after the time of *Ccm3* deletion (P1-P3). Retina tissues were harvested at P15. Retinas were subjected to whole-mount stained with Tie2 (red), p-Tie2 (green) and VE-cadherin (blue). Representative images of normal vessels (arrowheads) and CCM lesions (asterisks) are shown. n=6. Scale bars: 20 μm.

b. Tie2 inhibitor Rebastinib reduces α-SMA expression in brain. Vehicle or Rebastinib was subcutaneously injected into Ctrl and *Pdcd10*^{ECKO} mice from P4 after the time of *Ccm3* deletion (P1-P3). Brain sections were co-stained with CD31, α-SMA and NG2. Images were captured under STED microscopy. Representative images for WT normal vessels and CCM lesions in *Pdcd10*^{BECKO} mice are shown. n=3. α-SMA was undetectable in EC or pericyte of vehicle or Rebastinib-treated WT vessels (arrowhead). α-SMA^{+} cells were colocalized with NG2^{+} pericyte but not with CD31^{+} EC within a lesion of vehicle-treated *Pdcd10*^{BECKO} mice (arrows), but disappeared in Rebastinib-treated mice. n=6. Scale bar: 8 μm. Source data are provided as a Source Data file.

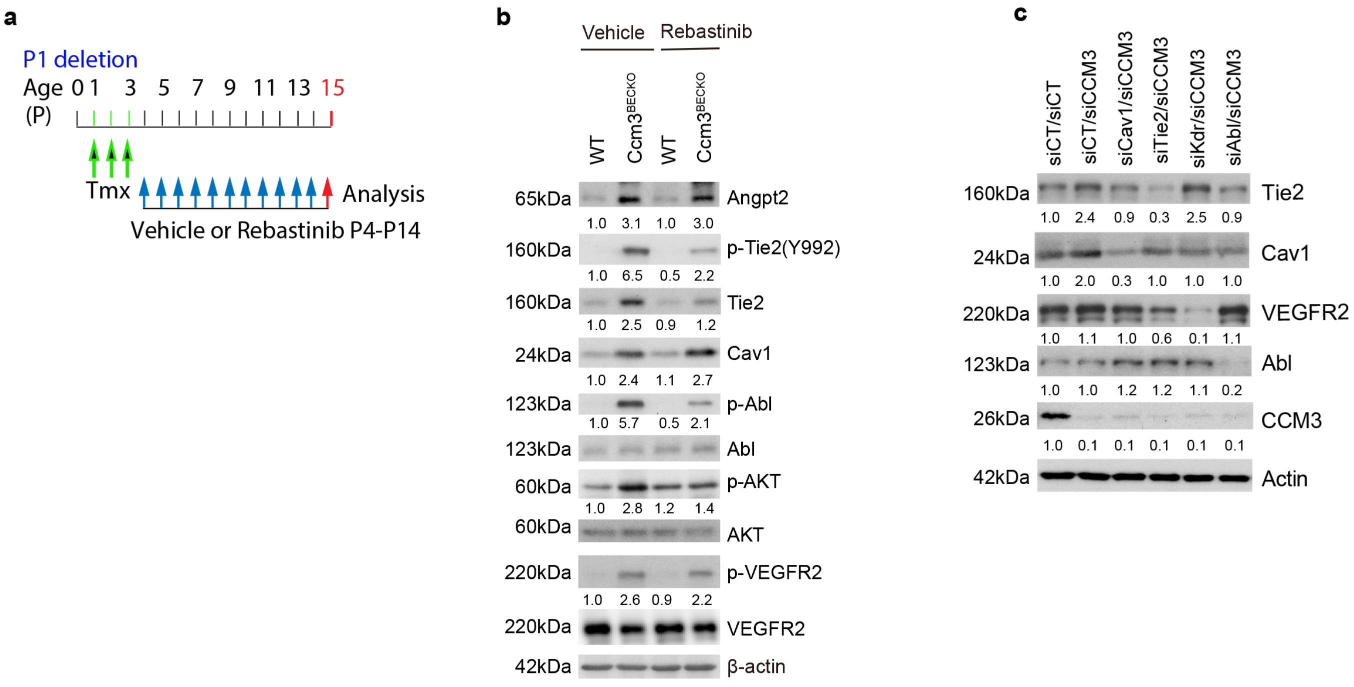

Supplementary Fig.11. Specific effects of Rebastinib on Tie2. a-b. Tie2 inhibitor Rebastinib blocks Cav1-Tie2 signaling in brain. **a.** A diagram for the therapeutic protocol**.** Tamoxifen injection at P1-P3 followed by administration of Rebastinib or vehicle control at P4-P14. Mice were sacrificed and analyzed at P15. **b**. Brain tissues were harvested at P15 and subjected to Western blotting with respective antibodies. Protein fold changes are indicated by taking vehicle-treated WT as 1.0. n=3.

c. Abl co-silencing, like Cav1 co-silencing, attenuates Tie2 expression. HBMVECs were transfected with Ctrl siRNAs, CCM3 siRNAs or co-silencing of CCM3 with Cav1, Tie2, Kdr or Abl for 72 h. The siRNA-transfected cells were subjected to Western blotting for proteins as indicated. Relative protein levels were quantified and fold changes are presented by taking Ctrl siRNA as 1.0. n=2. Source data are provided as a Source Data file.

Supplementary Table 1: Antibodies for immunostaining

Antibody name	Company	Cat #	Dilution
Angiopoietin-2, goat	R&D	AF7186	1:100
Caveolin-1, rabbit	Santa Cruz	sc-894	1:100
Caveolin-1, rabbit	Cell Signaling	3267	1:100
CCM3, rabbit	Abcam	ab180706	1:1000
CCM3, rabbit	Min lab		1:1000
CD31, rat	BD Pharmingen	553370	1:100
CD31, Armenian hamster	Millipore	MAB1398Z	1:400
Claudin-5, rabbit	Invitrogen	34-1600	1:100
Collagen IV, rabbit	Bio-Rad	2150-1470	1:300
Clathrin, rabbit	BD Pharmingen	610499	1:100
Endomucin, rat	Hycult Biotech	HM1108	1:300
Lamp1, rabbit	Cell Signaling	9091	1:100
NG2, rabbit	Millipore	AB5320	1:100
Rab11, rabbit	Cell Signaling	5589	1:100
SMA	Sigma	A2547	1:500
p-Tie2 (Tyr992), rabbit	Cell Signaling	4226	1:1000
p-Tie2 (Y992), rabbit	R&D	AF2720	1:100
Tie2, goat	R&D	AF762	1:100
Tie2, goat	R&D	AF313	1:100
Tie2, rabbit	Cell Signaling	4224	1:1000
VE-cadherin, rat	BD Pharmingen	555289	1:100
VE-cadherin, goat	Santa Cruz	sc-6458	1:100
ZO-1, rabbit	Invitrogen	61-7300	1:100
Ki67	Cell Signaling	9027	1:100
Alexa Flour 488 Phalloidin	Invitrogen	A12379	1 μg/mL
Alexa Flour 594 Isolectin GS-IB4 conjugate	Invitrogen	I21413	1 μg/mL
Alexa Flour 488 Donkey Anti-Rat IgG	Invitrogen	A21208	1 μg/mL
Alexa Flour 488 Donkey Anti-Goat IgG	Invitrogen	A11055	1 μg/mL
Alexa Flour 488 Donkey Anti-Rabbit IgG	Invitrogen	A21206	1 μg/mL
Alexa Flour 488 Donkey Anti-Mouse IgG	Invitrogen	A21202	1 μg/mL
Alexa Flour 594 Donkey Anti-Rat IgG	Invitrogen	A21209	1 μg/mL
Alexa Flour 594 Donkey Anti-Goat IgG	Invitrogen	A11058	1 μg/mL

Alexa Flour 594 Donkey Anti-Rabbit IgG	Invitrogen	A21207	1 μg/mL
Alexa Flour 594 Donkey Anti-Mouse IgG	Invitrogen	A21203	1 μg/mL

Supplementary Table 2: Primers for qRT-PCR:

Ccm3_FW:GCCCCTCTATGCAGTCATGTA
Ccm3_RV:AGCCTTGATGAAAGCGGCTC

Unc13b_FW:CTCTGCGTGCGCGTTAAAAG
Unc13b_RV: CAGGCGACTAATCTCAAACATGA

Tie2_FW:TCCGCTGGAAGTTACTCAAGA
Tie2_RV:GAACTCGCCCTTCACAGAAATAA

Actin_FW: CTGAGAGGGAAATCGTGCGT
Actin_RV: AACCGCTCGTTGCCAATAGT

18S rRNA_FW: GTAACCCGTTGAACCCCATT
18S rRNA_RV: CCATCCAATCGGTAGTAGCG
