# Supplementary material for: Caveolae-mediated Tie2 signaling contributes to CCM pathogenesis in a brain endothelial cell-specific Pdcd10-deficient mouse model
Source: Nat Commun. 2021 Jan 25;12:504. doi: 10.1038/s41467-020-20774-0 (PMC7835246; doi:10.1038/s41467-020-20774-0)
Supplement: Supplementary file 3 — Description of Additional Supplementary Files [file 41467_2020_20774_MOESM3_ESM.pdf]

Description of Additional Supplementary Files

File Name: Supplementary Movie 1-4
Description: **Visualization of CCM lesions in *Pdcd10*^{BECKO} mice by dynamic *in vivo* imaging (Movie for Fig.2c).** WT (Movie 1), *Pdcd10*^{BECKO} mice with mild (movie 2), moderate (movie 3) and severe lesions (movie 4) were visualized by Evans blue dye (EBD). Each movie was imaged from surface up to 100 µm deep in the cortex, and each image above the movie was Z stack.

File Name: Supplementary Movie 5-6
Description: **Visualization of increased caveolae in *Pdcd10*^{BECKO} brain lesions by tomography (Movie for Fig. S5c)**. Caveolae structure in representative electron tomography movies from WT (movie 5) and *Pdcd10*^{BECKO} mice (movie 6) are shown.
