# Supplementary material for: Author Correction: Caveolae-mediated Tie2 signaling contributes to CCM pathogenesis in a brain endothelial cell-specific Pdcd10-deficient mouse model
Source: Nat Commun. 2025 Jul 10;16:6352. doi: 10.1038/s41467-025-61617-0 (PMC12246070; doi:10.1038/s41467-025-61617-0)

Original Fig. 2

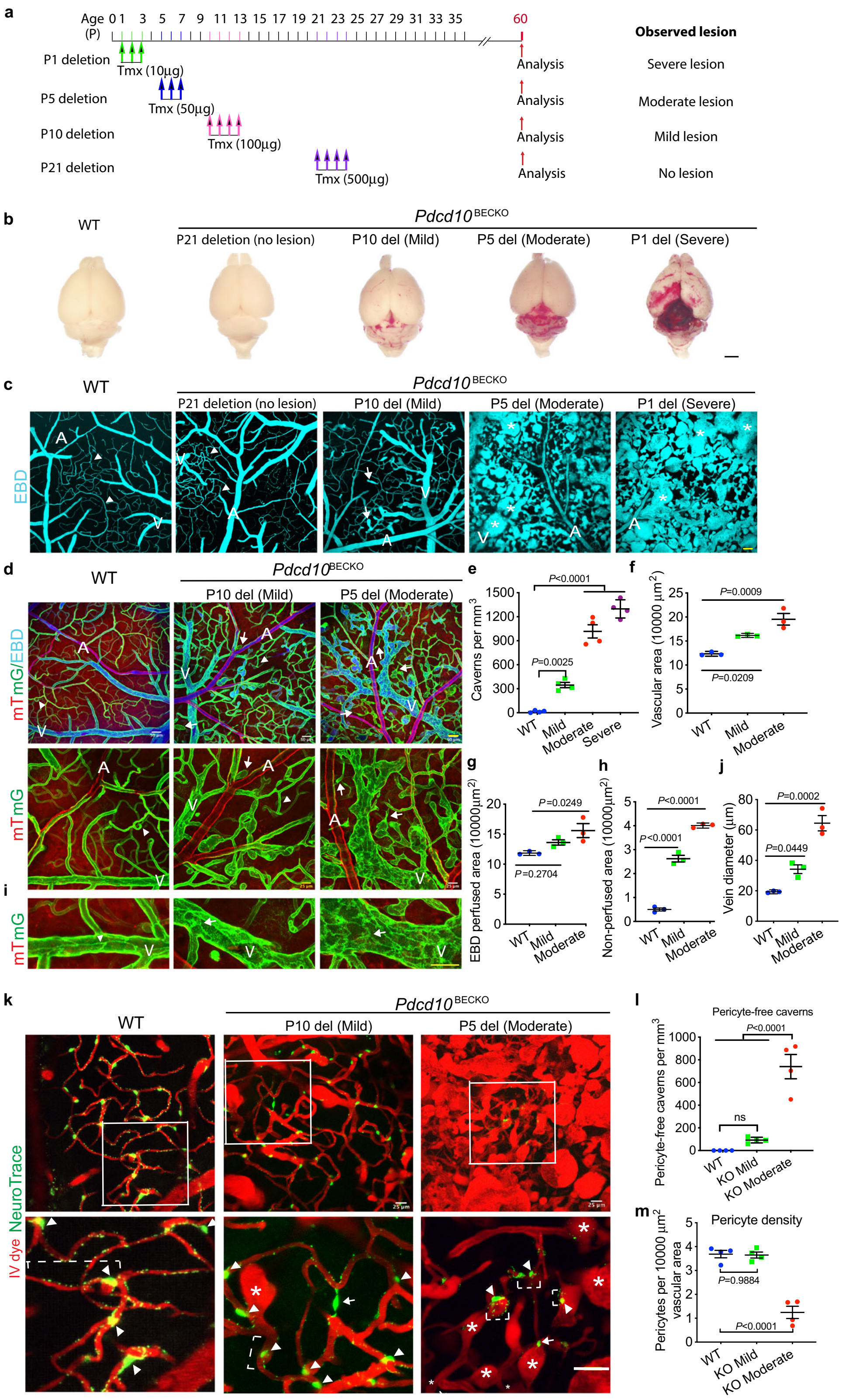

Original Fig. 5

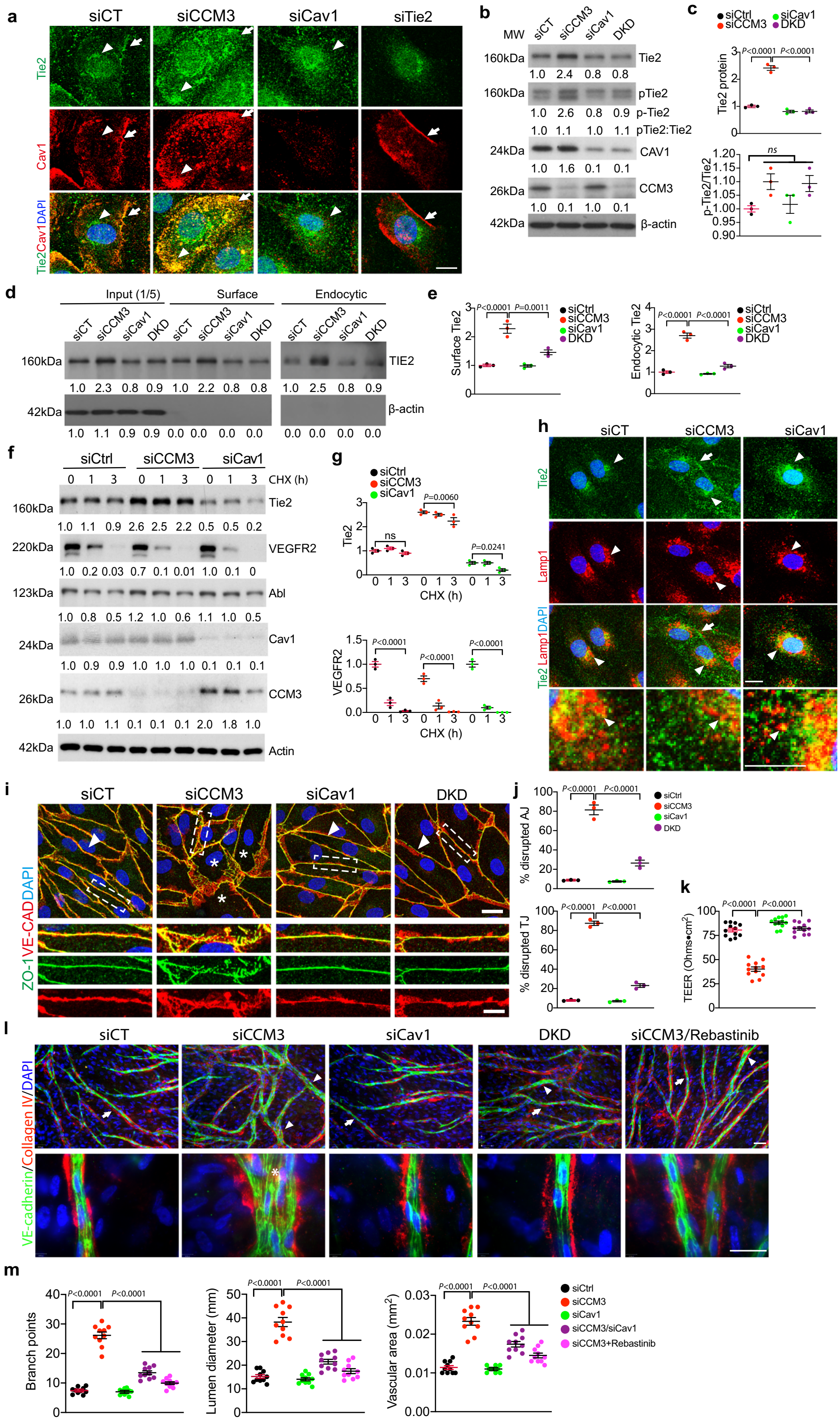

Original Fig. 6

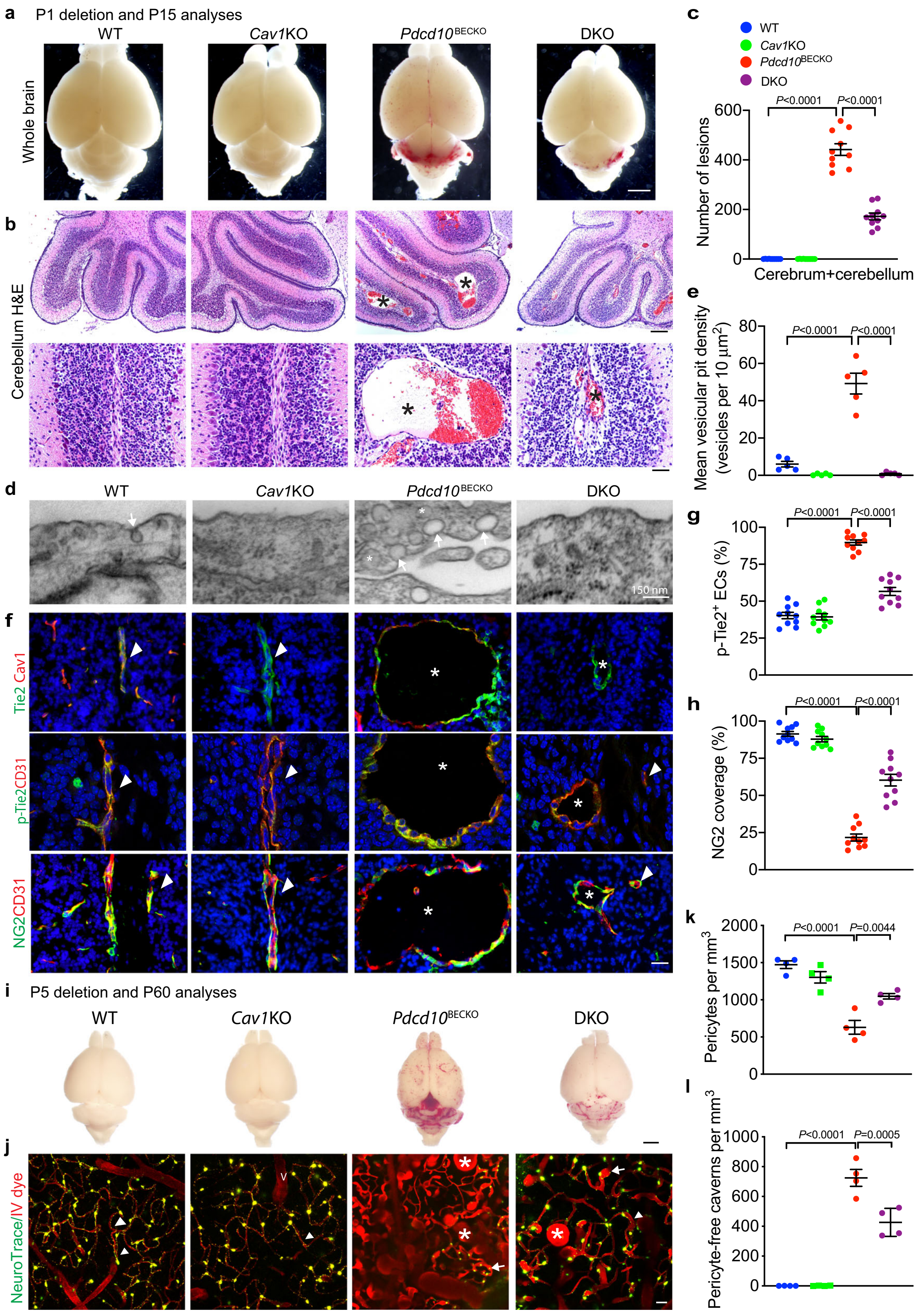

Original Supplementary Fig. 1

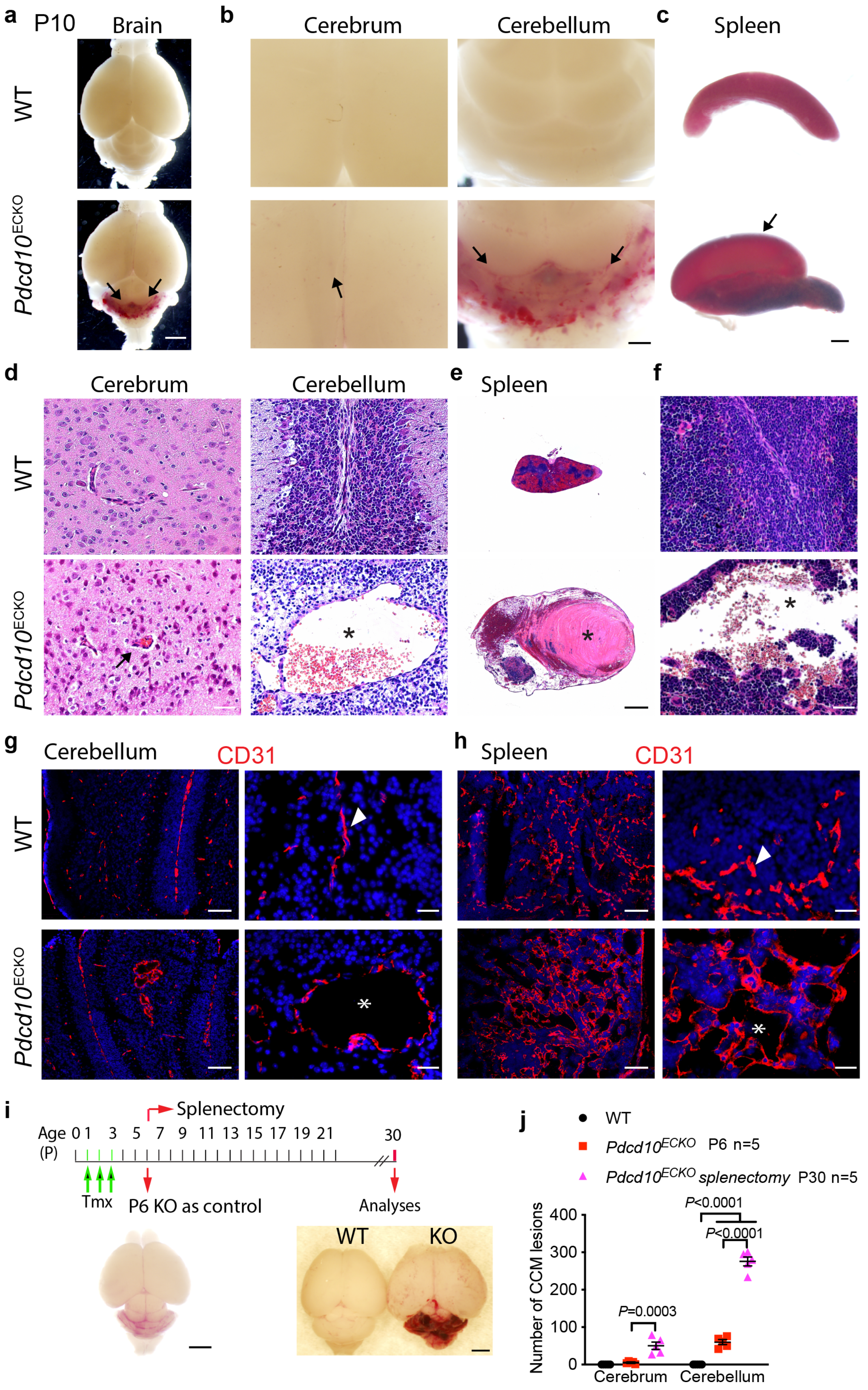

Original Supplementary Fig. 9

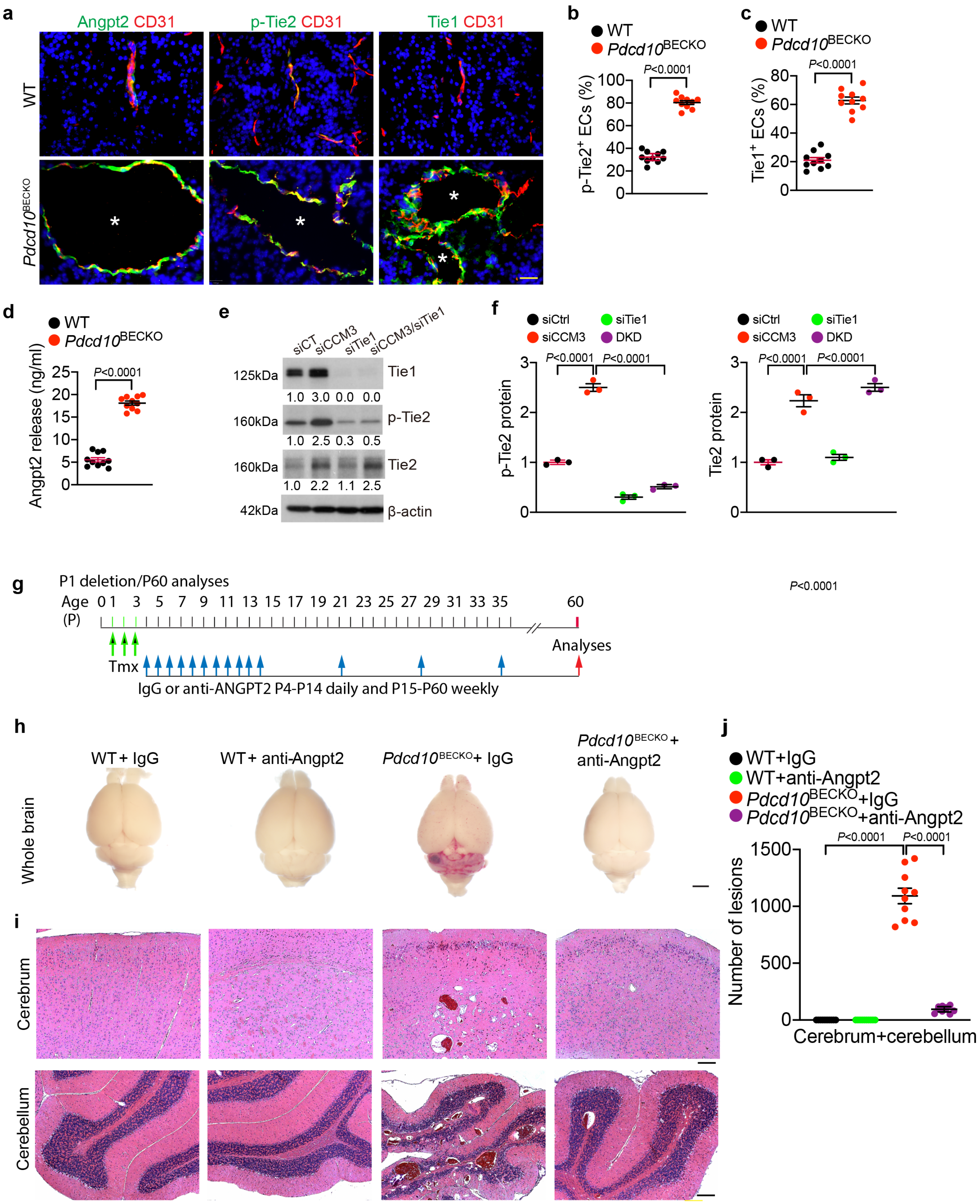

Original Supplementary Fig. 10

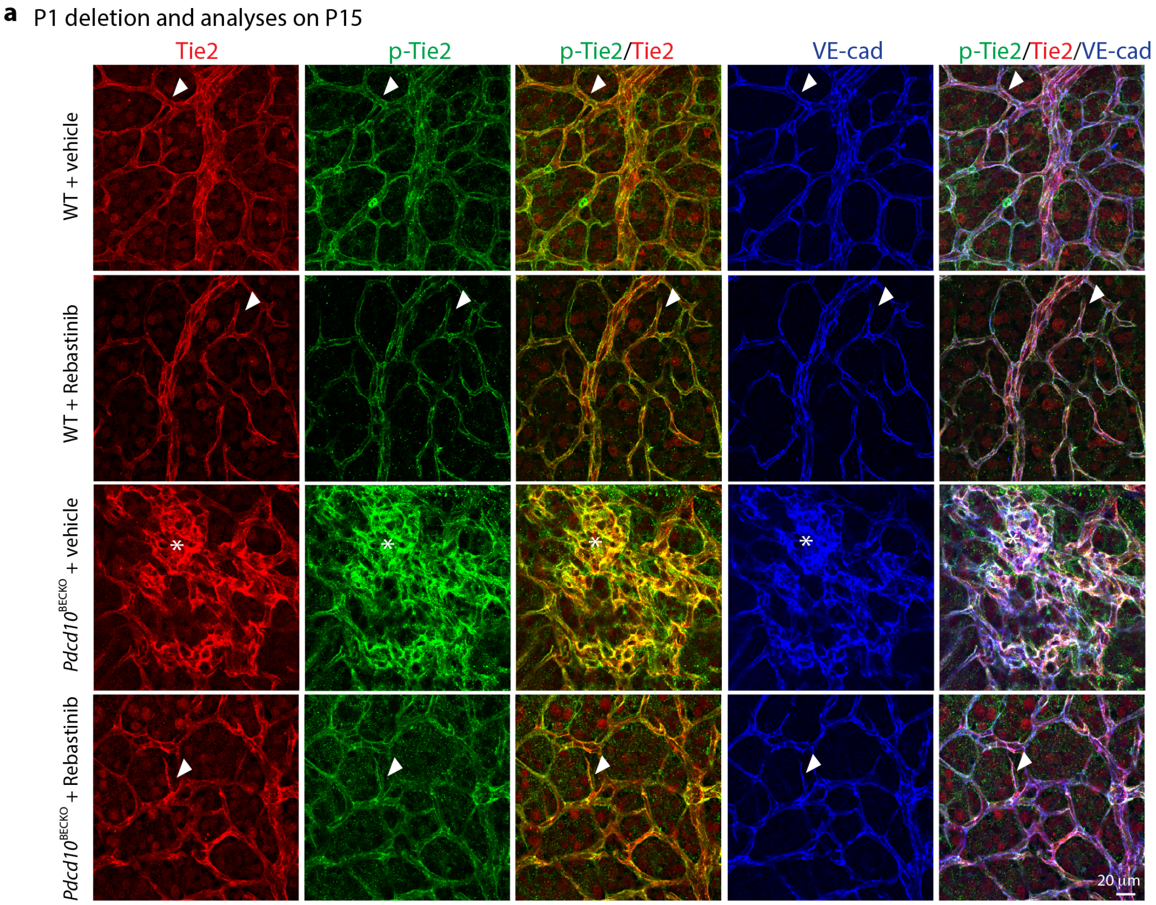

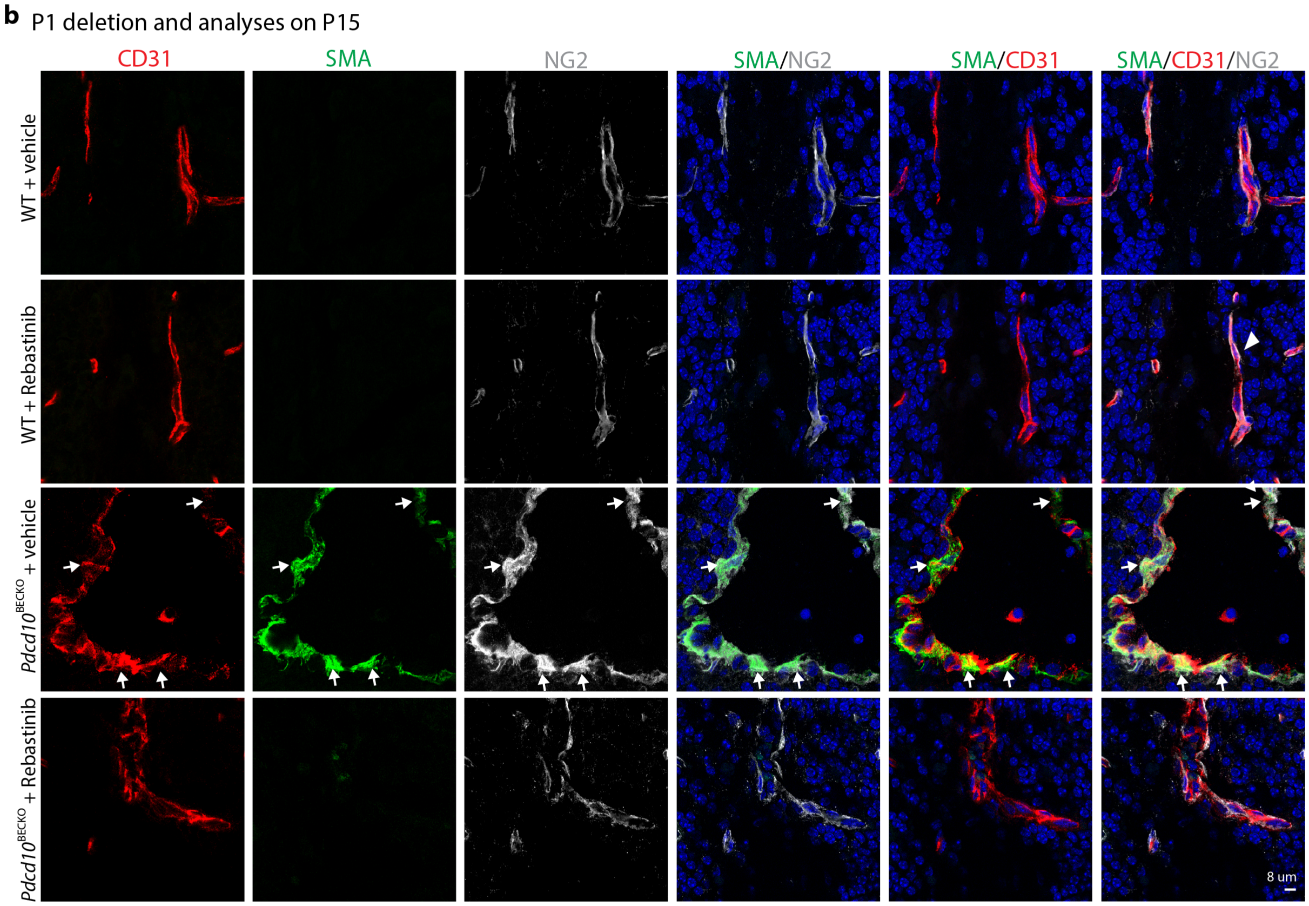

Supplement: Supplementary file 1 — Original Figs. 2, 5 and 6 and Supplementary Figs. 1, 9 and 10 [file 41467_2025_61617_MOESM1_ESM.pdf]
